# Correction: Composition design, electrical properties, and temperature stability in (1 − *x*)K_0.44_Na_0.56_Nb_0.96_Sb_0.04_O_3_-*x*Bi_0.45_La_0.05_Na_0.5_ZrO_3_ lead-free ceramics

**DOI:** 10.1039/d0ra90024a

**Published:** 2020-03-18

**Authors:** Jian Ma, Juan Wu, Bo Wu

**Affiliations:** Physics Department, Sichuan Province Key Laboratory of Information Materials, Southwest Minzu University Chengdu 610041 P. R. China majian33@hotmail.com; Sichuan Province Key Laboratory of Information Materials and Devices Application, Chengdu University of Information Technology Chengdu 610225 P. R. China wubo7788@126.com

## Abstract

Correction for ‘Composition design, electrical properties, and temperature stability in (1 − *x*)K_0.44_Na_0.56_Nb_0.96_Sb_0.04_O_3_-*x*Bi_0.45_La_0.05_Na_0.5_ZrO_3_ lead-free ceramics’ by Jian Ma *et al.*, *RSC Adv.*, 2018, **8**, 29871–29878.

The authors regret that an incorrect version of Fig. 4 was included in the original article. The correct version of Fig. 4 is presented below.

**Fig. 1:**
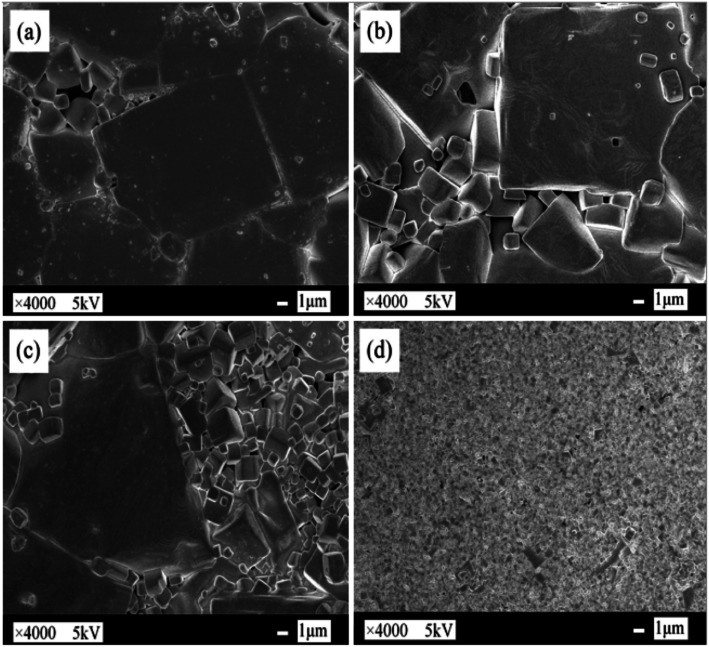
FE-SEM surface images of (1 − *x*)K_0.44_Na_0.56_Nb_0.96_Sb_0.04_O_3_-*x*Bi_0.45_La_0.05_Na_0.5_ZrO_3_ ceramics with (a) *x* = 0, (b) *x* = 0.020, (c) *x* = 0.040, (d) *x* = 0.060.

The Royal Society of Chemistry apologises for these errors and any consequent inconvenience to authors and readers.

## Supplementary Material

